# Transjugular intrahepatic portal shunt in the treatment of portal hypertension due to cirrhosis: single center experience

**DOI:** 10.1186/s12893-019-0659-5

**Published:** 2019-12-12

**Authors:** Yun Chen, Hanyu Qiu, Xiaomei Zhang

**Affiliations:** Department of Gastroenterology, Xiangya Hospital, Central South University, Changsha, 410000 Hunan China

**Keywords:** Liver cirrhosis, Portal hypertension, Gastrointestinal hemorrhage, Hepatic encephalopathy, Shunt, Stent dysfunction

## Abstract

**Aim:**

To investigate clinical efficacy of transjugular intrahepatic portal shunt (TIPS) for the treatment of cirrhotic portal hypertension.

**Methods:**

71 cases of patients with cirrhotic portal hypertension and esophageal and gastric variceal bleeding hospitalized from January 2014 to June 2017 were enrolled and treated with TIPS. The change of portal pressure and serum biochemical indexes before and after TIPS were compared, and re-hemorrhage rate, ascites incidence, complications, and survival rate were calculated.

**Results:**

71 patients (male/female 47/24, aged 29–77 years, average 48.9 ± 9.8 years) with cirrhotic portal hypertension received TIPS. The success rate of TIPS was 93% (66/71). During 1–24 months (mean 12.5 ± 7 months) follow-up of 66 patients, 61 cases survived and 5 cases died. The portal pressure decreased significantly from 40.48 ± 3.15 cmH2O to 23.59 ± 4.41 cmH2O after TIPS (*P* < 0.05). During the follow-up, the incidence of hepatic encephalopathy was 12.1%, the incidence of re-hemorrhage was 18.2%, and there were 4 cases of stent dysfunction, with 1 case of bare stent and 3 cases of dual stent.

**Conclusion:**

TIPS is an effective procedure for the treatment of cirrhotic portal hypertension complications, since it can reduce portal pressure and significantly alleviate ascites. Liver function is impaired in short-term after TIPS, but TIPS has no significant effect on liver function in middle-term.

## Introduction

Portal hypertension is an increase in the pressure within the portal vein, which carries the blood from the digestive organs to the liver [1]. Portal hypertension is one of important manifestations of decompensated cirrhosis. The main manifestation of portal hypertension are the opening of collateral circulation, esophageal and gastric variceal bleeding (EGVB), ascites, and splenomegaly [2, 3]. The incidence and severity of the complications of portal hypertension is the primary factor affecting hospitalization rate and mortality rate in patients with cirrhosis [4]. EGVB is the most common complication of portal hypertension, and the mortality in hospitalized patients is 30–50% [5].

Transjugular intrahepatic portal shunt (TIPS) was proposed by Rosch in 1969 [6]. With the improvement of this technique and the use of covered stent, TIPS has become an effective procedure for EGVB and refractory ascites that cannot be controlled by drugs and endoscopic treatment. During TIPS, a shunt is established in the liver between the caval venous system and the portal venous system in a minimally invasive manner, leading to significantly reduced portal vein resistance. TIPS is a key measure to reduce portal hypertension in patients with cirrhosis [7]. The 2007 American Association for the Study of Liver Diseases (AASLD) practice guidelines recommend that TIPS is preferably used to treat EGVB that cannot be controlled by drugs and endoscopic therapy [5]. So far, the short-term efficacy of TIPS treatment is positive, but the middle and long-term efficacy is controversial. In this study, we analyzed 71 patients with cirrhosis and portal hypertension who were treated with TIPS in our hospital, and compared short-term and middle-term efficacy and complications of TIPS.

## Subjects and methods

### Subjects

This study was approved by Ethics Committee of Xiangya Hospital of Central South University and all subjects provided informed consent. From January 2014 to June 2017, 71 patients with cirrhotic decompensated portal hypertension accompanied with EGVB and refractory ascites who were treated with TIPS at Xiangya Hospital of Central South University were enrolled.

The general clinical data of the patients are shown in Table 1, including 47 males and 24 females, and the age ranged from 29 to 77 years (average 48.9 ± 9.8 years). There were 46 cases of viral hepatitis cirrhosis (hepatitis B/hepatitis C 44/2), 6 cases of alcoholic cirrhosis, 7 case of mixed cirrhosis, 5 cases of autoimmune cirrhosis, 2 cases of Budd-Chiari syndrome, 4 cases of cryptogenic cirrhosis, 1 case of pancreatic portal hypertension. Based on liver function classification, 13 cases were Child-Pugh A, 44 cases were Child-Pugh B, and 14 cases were Child-Pugh C.

**Table 1:** General clinical data of 71 patients with TIPS

Variable	*n* = 71
Age	48.9 ± 9.8
Gender (male/female)	47/24
Cause of disease	
Viral hepatitis	46
Alcoholic liver disease	6
Autoimmune liver disease	5
mixed type	7
Budd-Chiari/cryptogenic/pancreatic Child-	2/4/1
Pugh classification [8]	
Class A/class B/class C	13/44/14
TIPS treatment reasons	
Hemorrhage/refractory ascites	63/8
TIPS surgery type (early/elective)	10/61
Stent type (bare stent/covered stent/dual stent)	12/10/44#

Note: The mean value expressed as χ ± SD, #: *n* = 66, 5 patients have failed surgery

### Procedure

Ultrasound, CT or MRI examinations were performed before procedure to understand the anatomy of intrahepatic branches of hepatic vein and portal vein, and provide auxiliary support for choosing the puncture direction, angle of hepatic vein to portal vein, and the blood flow state of portal vein. The liver and kidney function, coagulation function, blood type, blood transfusion and other related biochemical indices were measured before procedure.

After routine disinfection and local anesthesia, the right internal jugular vein was punctured, the guide wire was inserted into the inferior vena cava through the right atrium, the catheter was placed, and hepatic venous pressure was measured. According to preoperative imaging results, the right hepatic vein or the middle hepatic vein was selected as the initial puncture vein, and the proper puncture angle and plane were selected to enter the portal vein. The balloon was expanded and positioned, and the portal vein pressure was measured after the pigtail catheter was exchanged. According to the balloon dilatation pressure mark, the position and length of the punctured intrahepatic segment could be estimated. The RUS-100 puncture system was placed along the guide wire to the main portal vein, and the angiography was used to monitor blood flow and varicose veins of the main and branch of portal vein. The embolization treatment was performed with polyglycerol or anhydrous alcohol and a spring coil, and the embolization effect was determined by angiography. Under the guidance of the guide wire, a stent release system was placed between the hepatic vein and the portal vein puncture channel, an artificial shunt was established, and the stent was released after the position was determined. The portal vein pressure was measured again and the portal vein pressure significantly decreased, indicating that the procedure could be ended with compression hemostasis bandage.

After the procedure, routine monitoring was performed, paying attention to the patients’ vital signs, abdominal pain, consciousness, gastrointestinal hemorrhage, and blood oozing of puncture site. All patients received routine anticoagulant therapy (subcutaneous injection of low molecular weight heparin 3000 U), and appropriate anti-inflammatory treatment.

### Follow-up

Clinical follow-up was performed at 1 week, 1 month, 3 months, 6 months, 12 months, 18 months, and 24 months after TIPS. The follow-up included the general condition, hemorrhage, hepatic encephalopathy, ascites, etc., and liver function and blood routine tests. Some patients were examined by abdominal color Doppler ultrasound or DSA to determine the situation of the stent.

### Statistical analysis

All data were statistically analyzed using SPSS 25 software. The measurement data were expressed as χ ± SD. Preoperative and postoperative portal venous pressure, preoperative and postoperative hematology, and biochemical indices were analyzed by paired t test. The enumeration data were analyzed by χ^2^ test. *P* < 0.05 was considered significant.

## Results

### Outcomes of TIPS treatment

Of the 71 patients, 3 cases had failed stent placement due to their vascular anatomy; 2 cases had unstable circulation due to intraoperative hemorrhage, so the stent placement failed. The successful cases of TIPS were 66, and the success rate was 93%. During follow-up, 61 cases survived and five cases died, including one case died of abdominal thoracic hemorrhage 3 days after TIPS and 4 cases died of hepatic encephalopathy. Total 8 cases had hepatic encephalopathy, and 12 cases had recurrent hemorrhage. Total 4 cases had stent stenosis, 3 cases of them underwent the second TIPS and 1 case underwent hepatic vein balloon dilatation, and there was no hemorrhage during the follow-up period. The hematemesis and hematochezia of 53 patients with hemorrhage were effectively controlled after TIPS, and the short-term hemostatic rate was 100%. After TIPS, the therapeutic effect of 8 patients with refractory ascites was evaluated by color Doppler ultrasound and abdominal circumference measurement [12]. All 8 patients had obvious regression of ascites.

### Situation of stent use

12 cases used metal stents, 10 patients used Fluency covered stents, and 44 cases used dual stents. The diameter of all stents used in this study was 8 mm.

### Change of portal venous pressure before and after TIPS

As shown in Table 2, portal venous pressure decreased from 40.48 ± 3.15 cmH2O before TIPS to 23.59 ± 4.41 cmH2O after TIPS, and the difference was statistically significant (*P* < 0.05, paired t-test).

**Table 2:** Change in portal pressure before and after TIPS (χ ± SD, *n* = 66)

Measurement index (cmH2O)	Before operation	After operation		*P* value
portal pressure	40.48 ± 3.15	23.59 ± 4.41		0.000

### Changes of hematology and serum biochemical indices at 1 week before and 1 week after TIPS

As shown in Table 3, hemoglobin, serum albumin, alanine aminotransferase, and total bilirubin were significantly different before and after TIPS (*P* < 0.05), while the platelet, prothrombin time, international normalized ratio (INR), and aspartate aminotransferase were not significantly different before and after TIPS (*P* > 0.05).

**Table 3:** Changes in hematology and serum biochemical indices at 1 week before and 1 week after TIPS (χ ± SD, *n* = 65)

Serum index	Before operation (1 week)	After operation (1 week)	*P* value
Hemoglobin (g/l)	79.72 ± 2.32	84.70 ± 2.08	*0.006
Platelet (10^9/l)	65.14 ± 5.43	72.20 ± 9.14	0.271
Prothrombin time (s)	17.54 ± 0.38	18.03 ± 0.39	0.215
INR	1.40 ± 0.03	2.31 ± 0.88	0.300
Alanine aminotransferase(U/l)	34.40 ± 5.91	61.15 ± 8.29	*0.008
Aspartate aminotransferase (U/l)	59.50 ± 14.67	70.81 ± 7.11	0.447
Serum albumin (g/l)			
Total bilirubin (μmol/l)	31.89 ± 0.73 21.69 ± 1.57	31.49 ± 0.62 33.94 ± 4.38	*0.042 *0.007

Note: * Paired t-test, *P* < 0.05. *n* = 65, because one patient died of thoracic bloating and hemorrhage 3 days after TIPS

### Changes of liver function before and after TIPS

As shown in Table 4, the difference in serum albumin was statistically significant (*P* < 0.05) at 1 week, 3 months, and 12 months after TIPS, and the difference in alanine aminotransferase was statistically significant 1 week after TIPS (*P* < 0.05). The difference in total bilirubin was statistically significant at 1 week, 1 month and 3 months after TIPS (*P* < 0.05). However, the differences in serum albumin, alanine aminotransferase, aspartate aminotransferase, and total bilirubin were not statistically significant at 18 months and 24 months after TIPS (*P* > 0.05).

**Table 4:** Changes of liver function before and after TIPS (χ ± SD, *n* = 65)

Time	Serum albumin (g/l)	Alanine aminotransferase (U/l)	Aspartate aminotransferase (U/l)	Total bilirubin (μmol/l)
Before operation	31.89 ± 0.73	34.40 ± 5.90	58.08 ± 14.68	21.69 ± 1.56
1 week after TIPS	31.49 ± 0.62*	61.15 ± 8.29*	64.92 ± 6.97	33.53 ± 4.40*
1 month after TIPS	33.70 ± 0.99	34.32 ± 3.53	50.57 ± 4.62	27.62 ± 2.43*
3 months after TIPS	33.42 ± 0.57*	34.35 ± 3.50	46.57 ± 4.33	30.26 ± 3.19*
6 months after TIPS	31.84 ± 0.71	30.78 ± 3.18	40.65 ± 3.95	23.10 ± 2.05
12 months after TIPS	31.62 ± 0.69*	29.89 ± 3.24	44.53 ± 4.71	21.03 ± 1.33
18 months after TIPS	31.32 ± 0.60	27.72 ± 2.43	39.27 ± 3.27	21.29 ± 1.26
24 months after TIPS	32.02 ± 0.70	27.51 ± 2.45	39.43 ± 3.37	21.26 ± 1.47

Note: *: Compared with before operation, *P* < 0.05

### Relationship between hepatic encephalopathy and liver function and type of operation

As shown in Table 5, there were 2 cases of hepatic encephalopathy in the Child-Pugh A group, 5 cases of hepatic encephalopathy in the Child-Pugh B group, and 1 case of hepatic encephalopathy in the Child-Pugh C group, the difference was not statistically significant (chi-square test, χ^2^ = 0.472, *P* = 0.79). There was 1 case of hepatic encephalopathy in early operation group, and 7 cases of hepatic encephalopathy in elective operation group, the difference was not statistically significant (chi-square test, χ^2^ = 0.50, *P* = 0.823).

**Table 5:** Relationship between hepatic encephalopathy and liver function classification and type of operation (*n* = 66)

Group	Hepatic encephalopathy	None	Group	Hepatic encephalopathy	None
Child-Pugh class A	2	10	Early	1	9
Child-Pugh class B	5	36	Elective	7	49
Child-Pugh class C	1	12			

### Relationship between stent dysfunction and stent type

As shown in Table 6, there was 1 case of stent dysfunction in the bare stent group, 3 cases of dysfunction in the dual stent group but no stent dysfunction in the covered stent group, the difference in three groups was statistically significant (chi-square test, χ^2^ = 16.899, *P* = 0.001).

**Table 6:** Relationship between stent dysfunction and stent type (*n* = 66)

Stent type	Stent dysfunction	None
Bare stent	1	11
Covered stent	0	12
Dual stent	3	41

## Discussion

Through the establishment of the portacaval shunt, TIPS can significantly reduce portal pressure and control EGVB [8]. TIPS has become an effective treatment method for patients with EGVB and refractory ascites. In this study, our results showed that the hematemesis and hematochezia of 53 cases with hemorrhage were effectively controlled after TIPS, and the short-term hemostatic rate was 100%. In addition, the abdominal distension of 8 cases with refractory ascites was significantly improved after TIPS, and portal pressure significantly reduced after TIPS.

There is still no consensus on liver function changes after TIPS. Some studies suggested that liver function was impaired due to portal shunt [9, 10], while other study showed that liver function was improved after TIPS [11]. Because 2/3 of the liver blood supply comes from the portal vein and 1/3 comes from the hepatic artery, the establishment of TIPS shunt makes some portal vein blood directly enter the body vein, which reduces the perfusion of the portal vein to the liver and reduces metabolic function of hepatocytes, ultimately leading to hypoxia and necrosis of hepatocytes and impaired liver function. Combined with the iatrogenic liver injury caused by intraoperative operation, liver function may be impaired in early postoperative period [12]. However, hepatic blood flow can maintain a relative balance between the portal vein and the hepatic artery due to “hepatic artery buffer effect” [13]. After TIPS, as the hepatic artery blood supply gradually increases, the oxygen and nutrients required for liver metabolism increase, and liver function is recovered to a certain extent. In addition, the establishment of the shunt alleviates the congestion of the portal vein and reduces the lymphatic production of the liver. The establishment of the shunt also makes some portal vein blood directly enter the hepatic vein and the inferior vena cava, the cardiac output increases and the peripheral resistance decreases, thus the circulation and hepatic blood flow increase and can partially restore the liver function. Consistent with these speculations, in this study we found that TIPS puncture impaired liver function in the early stage but it had no significant effect on liver function in middle-term.

The complications of TIPS mainly include hepatic encephalopathy and stent dysfunction, and the incidence of hepatic encephalopathy after TIPS is 18–45% [14–17]. A meta-analysis found that old age, high Child-Pugh score, and history of hepatic encephalopathy were the main risk factors for hepatic encephalopathy after TIPS [18]. Our results showed that the differences in hepatic encephalopathy and Child-Pugh classification were not statistically significant. The stent dysfunction mainly includes stent stenosis, stent migration, stent rupture, and the rates of shunt stenosis and occlusion at 1, 2, and 5 years after TIPS were 5%/64, 33%/70, and 60%/85%, respectively [19]. Stent stenosis is mainly caused by excessive pseudointimal hyperplasia in the inner wall of the stent [20]. A retrospective study reported that the patency rates of covered stent and bare stent at 1 year and 2 years were 85.6%/46.6, 80.2%/18.6%, respectively, so the incidence of stent dysfunction was reduced by using covered stent [21]. In this study, during the 2-year follow-up of 66 patients with successful TIPS, 4 cases had stent stenosis, and the stenosis rate was 6%, lower than those reported previously, probably because more than 2/3 patients in this study used covered stents. In addition, our sample size was relatively small, which may cause the different results. The small sample size is a major limitation of this study. In addition, the follow-up was short, and we did not evaluate long-term outcomes after TIPS.

Due to iatrogenic mechanical injury and portal shunt after TIPS, liver function may be impaired to a certain extent in short-term, but it has no significant effect on liver function in middle-term. The main complications after TIPS are hepatic encephalopathy and stent stenosis. Therefore, it is necessary to evaluate hepatic encephalopathy history in patients before TIPS, and use covered stents to reduce the incidence of postoperative stent dysfunction.

## Conclusion

TIPS is an effective procedure for the treatment of cirrhotic portal hypertension complications, since it can reduce portal pressure and significantly alleviate ascites. Liver function is impaired in short-term after TIPS, but TIPS has no significant effect on liver function in middle-term.

## Data Availability

All data and materials are available upon reasonable request to the corresponding author.

## Abbreviations

EGVB: Esophageal and gastric variceal bleeding
TIPS: Transjugular intrahepatic portal shunt
